# Genome-wide profiling of copy number alterations in triple-negative breast cancer identifies a region at 19p13 associated with lymph node metastasis

**DOI:** 10.1186/1753-6561-7-S2-P24

**Published:** 2013-04-04

**Authors:** Felipe Fidalgo, Amanda Gonçalves, Tatiane Rodrigues, Alex Fiorini, Dirce Maria Carraro, Carla Rosenberg, Ana Cristina Victorino Krepischi

**Affiliations:** 1 CIPE, AC Camargo Hospital, São Paulo, Brazil; 2 Institute of Biosciences, University of São Paulo, Brazil

## Background

The acquisition of somatic alterations (point mutations/chromosomal rearrangements) underlies the hallmarks of cancer, generating genetic diversity that drives tumorigenesis. Advances in the study of cancer genomes revealed in solid tumors a complex pattern of copy number alterations (CNA), structural rearrangements, and aneuplodies. Breast cancer (BC) is the most common malignancy in females, being the leading cause of death by cancer. This heterogeneous disease is not fully understood yet; however, genomic studies have identified unique CNA patterns in different BC subtypes. Regarding the subtype triple-negative (**TN**; estrogen and progesterone receptors, and HER2 **negative** expression levels), only limited data are available on which genes or chromosome regions are involved in its initiation and progression.

## Material and methods

We assessed the genomic profile of CNAs in 16 triple-negative breast carcinomas (TNBC), aiming to identify genomic markers related to lymph node status that could be of clinical importance. The array-CGH survey were conducted on primary TNBC samples, obtained from patients with (8) and without (8) lymph node metastasis at diagnosis. Data was obtained using a 60K oligoarray (OGT), with average probe spacing ~50kb, and analysis was performed on the software Nexus Copy Number 6.0 (Biodiscovery).

## Results and discussion

The most prevalent chromosomal alterations were losses at 3p, 4q, 5q, 8p, 13q, 18, and 21q; additionally, gains on 1q, 3q, 8q, 10p, 17q e 19 were frequent events. However, although these CNAs have been detected in >50% of all samples, most of them exhibited low log2 ratios values, indicating that they were present in mosaic. Notably, TNBC from patients with lymph node metastasis exhibited a slightly distinctive pattern of genomic alterations, mainly characterized for a significantly increased frequency of low amplitude losses at 13q and 18q, and gain at 19p. The most relevant CNA associated with TNBC positive for lymph node metastasis was the gain of a small segment at 19p13.11 (660kb), harboring at least 20 RefSeq genes (gain detected in 5/8 TNBC positive and 0/8 negative for lymph node metastasis).

## Conclusion

The recurrence of specific CNAs in cancer indicates sites likely harboring genes whose copy number changes favors neoplastic progression. Therefore, recognizing specific patterns of genomic alterations associated to lymph node metastasis in primary TNBC can provide clues on the mechanisms driving its progression. In a progressive model of tumor evolution we can propose a link between the somatic acquisition of the 19p13.11 gain in TNBC and the occurrence of lymph node metastasis. It is interesting to mention that a recent study (*Stevens et al*, *Cancer Res 2012*) discloses a 19p13.11 SNP variant associated with risk of TNBC; our finding reinforces the role of this genomic region for this BC subtype.

## Financial support

FAPESP and CNPq.

